# Systems Biology and Peptide Engineering to Overcome Absorption Barriers for Oral Peptide Delivery: Dosage Form Optimization Case Study Preceding Clinical Translation

**DOI:** 10.3390/pharmaceutics15102436

**Published:** 2023-10-09

**Authors:** Puneet Tyagi, Chandresh Patel, Kimberly Gibson, Fiona MacDougall, Sergei Y. Pechenov, Sarah Will, Jefferson Revell, Yue Huang, Anton I. Rosenbaum, Kemal Balic, Umar Maharoof, Joseph Grimsby, J. Anand Subramony

**Affiliations:** 1 Dosage Form Design and Development, BioPharmaceuticals R&D, AstraZeneca, Gaithersburg, MD 20878, USA; 2 BDD Pharma, Glasgow G4 0SF, UK; 3 Bioscience Metabolism, Cardiovascular, Renal and Metabolism, Biopharmaceuticals R&D, AstraZeneca, Gaithersburg, MD 20878, USAjoseph.grimsby@regeneron.com (J.G.); 4 Discovery Sciences, BioPharmaceuticals R&D, AstraZeneca, Cambridge CB2 0AA, UK; 5 Integrated Bioanalysis, Clinical Pharmacology & Safety Sciences, R&D, AstraZeneca, San Francisco, CA 94080, USAanton.rosenbaum@astrazeneca.com (A.I.R.); 6 Clinical Pharmacology and Quantitative Pharmacology, Clinical Pharmacology & Safety Sciences, R&D, AstraZeneca, San Francisco, CA 94080, USA; k.balic@yahoo.com; 7 Biologics Engineering, Oncology R&D, AstraZeneca, Gaithersburg, MD 20878, USA

**Keywords:** oral delivery, peptides, permeation enhancers, controlled release, formulation optimization, MEDI7219

## Abstract

Oral delivery of peptides and biological molecules promises significant benefits to patients as an alternative to daily injections, but the development of these formulations is challenging due to their low bioavailability and high pharmacokinetic variability. Our earlier work focused on the discovery of MEDI7219, a stabilized, lipidated, glucagon-like peptide 1 agonist peptide, and the selection of sodium chenodeoxycholate (Na CDC) and propyl gallate (PG) as permeation enhancer combinations. We hereby describe the development of the MEDI7219 tablet formulations and composition optimization via in vivo studies in dogs. We designed the MEDI7219 immediate-release tablets with the permeation enhancers Na CDC and PG. Immediate-release tablets were coated with an enteric coating that dissolves at pH ≥ 5.5 to target the upper duodenal region of the gastrointestinal tract and sustained-release tablets with a Carbopol bioadhesive polymer were coated with an enteric coating that dissolves at pH ≥ 7.0 to provide a longer presence at the absorption site in the gastrointestinal tract. In addition to immediate- and enteric-coated formulations, we also tested a proprietary delayed release erodible barrier layer tablet (OralogiK^TM^) to deliver the payload to the target site in the gastrointestinal tract. The design of tablet dosage forms based on the optimization of formulations resulted in up to 10.1% absolute oral bioavailability in dogs with variability as low as 26% for MEDI7219, paving the way for its clinical development.

## 1. Introduction

Oral delivery of proteins and peptides has been gaining predominance since the 1970s, when it was observed in the clinic that intact macromolecules can be absorbed from the gastrointestinal (GI) tract. Despite the challenges posed by the barrier mechanisms within the GI tract, oral delivery of macromolecules can provide many benefits to patients over injections [1]. Efforts have continued over the years in both academia and industry to achieve absorption of large molecules in therapeutically meaningful doses orally that could potentially change the standard of care. To date, most approaches to the oral delivery of protein therapeutics have relied on the protection of the peptide and protein molecules from enzymatic degradation in the GI tract. Extensive structural alterations have been made to these macromolecules in general, and to peptides in particular, to obtain valuable information on their degradation in vivo [2].

In general, orally delivered peptides and proteins are absorbed via paracellular and transcellular routes. During paracellular transport, the opening of the tight junctions facilitates the entry of proteins and peptides. This route has been widely studied due to the availability of chemical permeation enhancers, many of which have reached the clinic for peptides such as calcitonin [3] and octreotide [4]. During transcellular transport, the molecule diffuses through lipid membranes or uses carrier- or receptor-mediated transport mechanisms. The transcellular route is of less interest than the paracellular route, owing to the considerable effort that is required to explore formulation development for this route [5]. Furthermore, transcellular transport is not expected to be the main contributor to improve delivery of hydrophilic drugs such as peptides [6].

Various permeation enhancer dependent oral peptide delivery formulations have reached the clinic, out of which insulin and glucagon-like peptide 1 (GLP-1) are predominant (Table 1). Other peptides that have advanced to the clinic in an oral presentation include parathyroid hormone, calcitonin, and octreotide.

**Figure 1:**
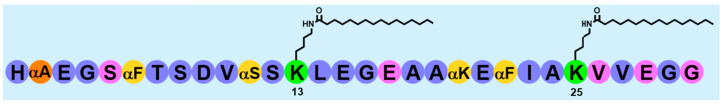
Chemical structure of MEDI7219. For details about the chemical structure, please see [12].

Here we describe the comprehensive optimization of the oral tablet formulations of a glucagon-like peptide 1 (GLP-1) receptor agonist a molecule, MEDI7219, (Figure 1) using various controlled-release technologies to enable absorption at the site of interest. In an earlier publication, we described our work on the early preclinical development of oral MEDI7219 [12]. GLP-1 RAs provide clinically viable reductions in elevated blood sugar, glycated hemoglobin, body weight, blood pressure, and cardiovascular risk in patients with type 2 diabetes and obesity [13]. MEDI7219 was stabilized against gastrointestinal peptidases and proteases by the introduction of unnatural amino acids at protease degradation hotspots. Further, side-chain lipidation was added to MEDI7219 to increase its half-life via an albumin reversible binding mechanism. MEDI7219 has been tested in in vitro and in vivo studies for receptor binding and pharmacodynamic effects in rodent models. The MEDI7219 stabilized lipidated peptide has good solubility and great proteolytic stability. Furthermore, potency in cell cultures and in rodent obesity and diabetes models was comparable to semaglutide [12]. To increase systemic bioavailability upon oral administration, we have identified Na CDC and a combination of Na CDC and PG permeation enhancers in Caco-2 screens and confirmed their effect in vivo after intraduodenal administration in rats. In vivo administration of a non-optimized tablet formulation of this combination in dogs resulted in bioavailability of approximately 4%, as well as decreased blood glucose excursion and reduced food consumption in an HF/HF dog model [12].

We conducted in vivo studies to achieve the additional optimization of MEDI7219 tablets in beagle dogs. The aim of these studies was to maximize MEDI7219 exposure while minimizing pharmacokinetic variability and to use minimal amounts of peptide and permeation enhancers while still achieving pharmacologically relevant MEDI7219 exposure. Based on the results of our prior site-of-absorption studies [12], we designed tablet formulations to deliver the peptide to the optimal absorption site by using a pH-specific enteric coating and immediate-release and time delayed-release formulations. Our results demonstrate the optimization of peptide content and the ratio of permeation enhancers in the tablet to maximize bioavailability and minimize the use of permeation enhancers in each tablet. To the best of our knowledge, this is a first-of-its-kind study of this magnitude that compared three distinctive release profiles of a target peptide at different sites of absorption.

## 2. Materials and Methods

MEDI7219 was manufactured by Bachem (Bubendorf, Switzerland). The permeation enhancers Na CDC and PG were purchased from Sigma-Aldrich (St. Louis, MO, USA). Eudragit polymers and PlasACRYL HTP 20 were purchased from Evonik (Allentown, PA, USA). Mucoadhesive polymer Carbopol 71G was purchased from Lubrizol (Wickliffe, OH, USA). HPMC Hypromellose K4M and Acryl-eze 93A were purchased from Colorcon (Harleysville, PA, USA). Glyceryl behenate was purchased from Gattefossé (Eschbach, Germany). Hydrogenated castor oil was purchased from BASF (Ludwigshafen, Germany). Low-substituted hydroxypropyl cellulose (LH-21 and LH-32) was purchased from Shin-Etsu (Tokyo, Japan). Simethicone was purchased from Basildon (Abingdon, UK). All other ingredients were purchased from Sigma-Aldrich. The OralogiK time-delayed tablets were designed and manufactured by BDD Pharma (Scotland, UK).

## 3. Preparation of Peptide Molecules

MEDI7219 was prepared by solid-phase peptide synthesis as the C-terminal carboxamide on NovaSyn TGR resin (initial substitution, 0.24 mmol/g). All peptide couplings were performed at ambient temperature for 45 min, using HCTU (O-(1H-6-chlorobenzotriazole-1-yl)-1,1,3,3-tetramethyluronium hexafluorophosphate)–diisopropylethylamine (DIPEA) in *N*-methylpyrrolidone (NMP)– dimethylformamide (DMF). Residues immediately following incorporation of α-methyl analogues were always coupled twice to ensure full incorporation. Following acylation, capping of residual functionality was performed over 10 min using a solution of acetic anhydride and pyridine in DMF. The fluorenylmethoxycarbonyl (Fmoc)-protecting group was deblocked by using piperidine in DMF (20% *v*/*v*) at ambient temperature for 2 × 10 min each. The N-terminal histidine was incorporated as the Boc-His(Trt)-OH to simplify cleavage. Fmoc-L-Lys(Mmt)-OH was incorporated into positions 13 and 25 (Tyr19 and Trp31 in hGLP-1 7-36, respectively) to allow for side-chain functionalization. Upon complete assembly of the peptide, the Mmt-protecting groups were selectively removed by manual treatment of the resin with 1% trifluoroacetic acid and 2% triisopropyl silane (TIPS) in dichloromethane (10 × 1 min, 20.0 mL/g). The acidified resin was quenched with 5% DIPEA-NMP, and the exposed lysine ε-amino functions were functionalized manually prior to peptide cleavage. The crude peptide was liberated from the resin by treatment with a cocktail consisting of trifluoroacetic acid (95% *v*/*v*), TIPS (2.5% *v*/*v*), and water (2.5% *v*/*v*) with agitation (3 × 1 h at ambient temperature). Purification of the crude bis-lipidated peptide was performed by dissolving the crude peptide in 0.1 M ammonium bicarbonate solution (1:5 acetonitrile/water, *v*/*v*; pH 8.0), which was filtered prior to sample loading (30 × 250 mm X-Bridge C18 Beh column; Waters, Milford, MA, USA). Purified peptides were characterized by liquid chromatography–mass spectrometry, using a Mass Lynx 3100 platform (Waters, Milford, MA, USA) eluting over an X-Bridge C18 Beh column (Waters), with detection by both ultraviolet absorption at 210 nm and electrospray ionization using a 3100 mass detector (Waters, Milford, MA, USA).

## 4. Formulation Preparation

### 4.1. Enterically Coated Immediate-Release Tablets (Formulations 1–16)

**Figure 2:**
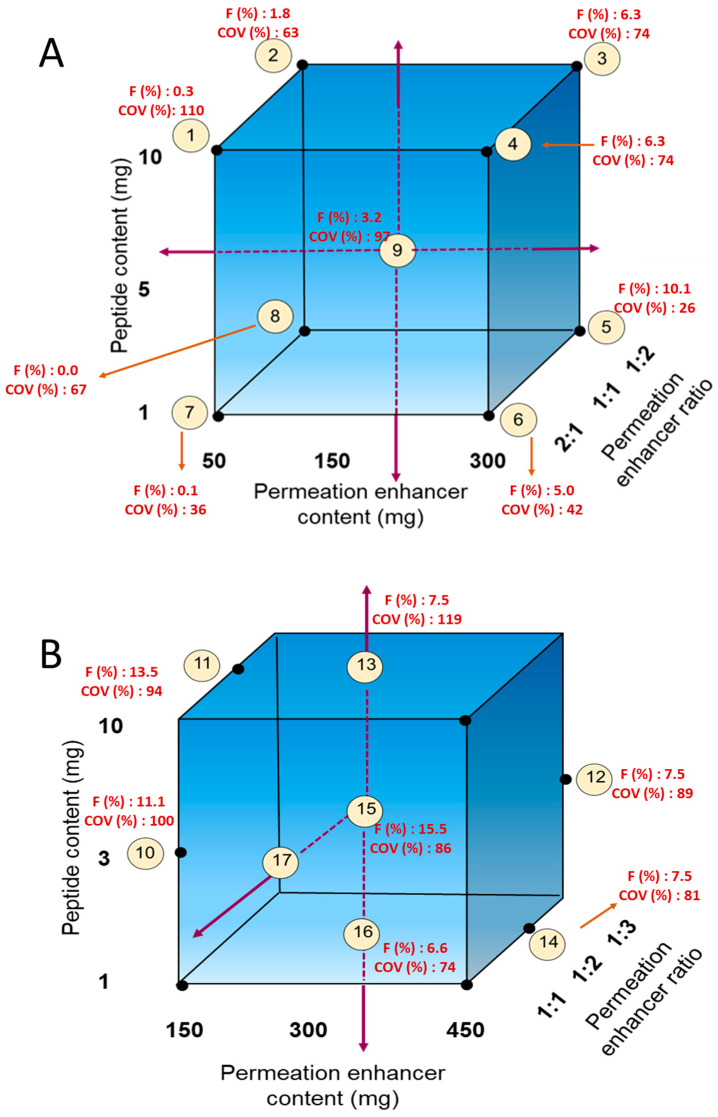
Formulations for optimization of immediate-release tablet formulations. (**A**,**B**) correspond to the cohort 1 and 2 in Table 2. Bioavailability (F%) and pharmacokinetic variability at C_max_ (COV%) is shown in red near each formulation.

The cores for the enterically coated immediate-release (EC-IR) tablets were prepared by dry granulation. For the first level of optimization (Figure 2A), 1, 5, and 10 mg of MEDI7219 and 50–450 mg of permeation enhancers in ratios of 1:1, 1:2, and 2:1 were evaluated. Filler, lubricant, and a glidant were added. All ingredients needed to make the required number of tablets were weighed, passed through a sieve, and blended. The blend was then mixed in a Turbula mixer (GlenMills, Clifton, NJ, USA). Dry granulation was performed by compressing the blend with a pellet machine. After compression for dry granulation, the compressed slug was passed through a sieve with a 1-mm opening to prepare granules. Additional Aerosil (Evonik) and Pruv (JRS Pharma) silica lubricants were mixed with the granules in a Turbula mixer. Tablets were compressed on an RD-10 tablet press machine (Natoli, Saint Charles, MO, USA) to a hardness of 50 N.

The second level of optimization (Figure 2B) was based on results obtained from the first optimization. In this second level, we tested a peptide content of 3 mg (in addition to 1 and 10 mg), increased the permeation enhancer content to 450 mg, and also tried a permeation enhancer ratio of 1:3.

### 4.2. Enterically Coated Controlled-Release Tablets (Formulations 17–22)

For the preparation of the enterically coated controlled-release (EC-CR) tablets, the controlled-release polymer (Carbopol or Eudragit RSPO) was added to the core tablet blend prior to granulation. Each tablet contained 1–8 mg of MEDI7219, 300 mg of permeation enhancers, added filler, lubricant, and a glidant, resulting in tablets weighing 245 mg. Enteric coating with Eudragit polymer suspensions was performed using a Vector LDCS3 pan coater (Freund-Vector, Marion, IA, USA) to achieve a weight gain in coated tablets of 12.5%. Cores were enterically coated with an Eudragit enteric coating dispersion (pH 5.5) to target the proximal small intestine and another Eudragit enteric coating (pH 7.0) to target the distal small intestine and proximal colon.

### 4.3. OralogiK Erodible Barrier Layer (EBL) Tablet (Formulations 23 and 24)

**Figure 3:**
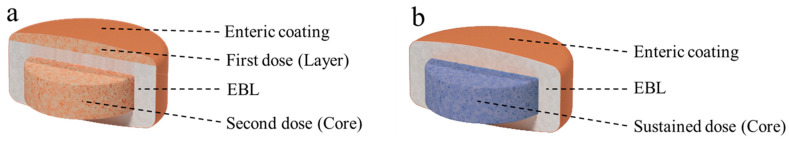
Schematic diagram of different layers of tablet. (**a**) OralogiK Formulation 1; (**b**) OralogiK Formulation 2.

Two different erodible barrier layer (OralogiK) tablet formulations containing a dose of 0.5 mg MEDI7219 per tablet were prepared, with the structures as depicted in Figure 3. Formulation 23 was designed to deliver the first dose following gastric emptying and dissolution of the enteric coat, followed by gradual erosion of the Oralogik delay layer, with a second dose that is released at around 3 h after gastric emptying as the tablet is expected to enter the ascending colon.

Formulation 24 had a sustained-release core that would only begin to disperse once the tablet had emptied from the stomach, the enteric coat dissolved, and the Oralogik delay layer eroded, with an expected onset of release around 4 h after gastric emptying (targeting the proximal colon), followed by sustained release for around 2 h.

The delayed-release cores for both EBL formulations were manufactured by blending MEDI7219, enhancers, and excipients, followed by direct compression. The core for formulation 23 contained 0.25 mg MEDI7219, along with microcrystalline cellulose, croscarmellose sodium, magnesium stearate, and the permeation enhancers Na CDC and PG and weighed a total of 125 mg. The sustained-release core for formulation 24 contained 0.5 mg MEDI7219, hydroxypropyl methylcellulose K4M, microcrystalline cellulose, croscarmellose sodium, and magnesium stearate and weighed 200 mg.

The EBL for both formulations was prepared by blending glyceryl behenate, hydrogenated castor oil, and low-substituted hydroxypropyl cellulose, followed by melt-mixing, cooling, and milling in an oscillating granulator.

The tablet cores for Formulations 23 and 24 were compression coated with a layer of this EBL to afford the target delayed-release profiles, and for Formulation 23, an additional immediate-release layer containing 0.25 mg MEDI7219 (providing a total dose of 0.5 mg) and composed of the same materials as the core was compressed at the top of the tablet. Formulations 23 and 24 were then coated with a plasticized enteric coat (Acryl-eze 93A) to a weight gain of 10–12%. in a mini-coater (Caleva, Dorset, UK).

## 5. In Vivo Studies

Animal studies were conducted at Charles River Laboratories (formerly MPI Research, Mattawan, MI, USA). Study protocols were approved by the Institutional Animal Care and Use Committee at Charles River Laboratories (Mattawan, MI, USA) and were in compliance with national laws and regulations ensuring humane use and care of laboratory animals and the AstraZeneca Animal Welfare and Bioethics policies.

In vivo pharmacokinetic studies were performed in male beagle dogs. Animals orally dosed with tablets (n = 5–7/group) were fasted for up to 8 h prior to dosing and food was returned approximately 4–5 h post-dose. Animals treated IV (n = 3/group) with MEDI7219 were not fasted. Tablet formulations containing ≥300 mg permeation enhancer were dosed as two tablets. OralogiK Erodible barrier layer tablets were dosed as two tablets per administration.

Blood was collected for plasma exposure at pre-dose, 0.083, 0.25, 0.5, 1, 2, 4, 8, 24, 48, 72, and 96 h after MEDI7219 intravenous (IV) dosing. In the groups receiving oral tablet formulations, blood was collected for plasma exposure at multiple timepoints that may include pre-dose, 0.25, 0.5, 1, 1.5, 2, 2.5, 3.5, 4.5, 8, 24, 48, 72, and up to 96 h after MEDI7219 dosing.

## 6. Quantification of Plasma Concentrations of Peptides

Fifty- or seventy-microliter aliquots of dipotassium ethylenediaminetetraacetic acid plasma samples were precipitated with 75% (*v*/*v*) acetonitrile (Sigma-Aldrich) in water (J.T. Baker, Phillipsburg, NJ, USA) and centrifuged at 2000× *g*. The supernatant was dried under nitrogen at 60°C and then cooled. Samples were reconstituted in 20% (*v*/*v*) acetonitrile in water. The extracted samples were separated with an Acquity UPLC BEH C18 column (2.1 × 100 mm; Waters) on a Nexera UHPLC (Shimadzu, Kyoto, Japan) at 60°C with a flow rate of 0.7 mL/min and detected using either a TripleTOF 6600 mass spectrometer (SCIEX, Framingham, MA, USA) operating in full-scan MS2 positive-ion mode, a 5500 mass spectrometer (SCIEX) operating in multiple reaction monitoring positive-ion mode, or a TQS Vantage mass spectrometer (Thermo Fisher, Waltham, MA, USA) operating in multiple reaction monitoring mode. Gradient separation was performed with water and 0.2% formic acid (Thermo Fisher) as mobile phase A and acetonitrile with 0.2% formic acid as mobile phase B. The method was qualified for a quantification range of 1–1000 ng/mL with accuracy and precision of ±20% except at the lower limit of quantification, when accuracy and precision were ±25%. Plasma concentrations were subjected to noncompartmental analysis consistent with the route of administration, using Phoenix WinNonlin software (version 7.0; Certara, Princeton, NJ, USA).

## 7. Results

### Optimization of Tablet Formulations in Dogs

We designed MEDI7219 tablet formulations based on our previous site-of-absorption studies [12] and optimized the composition of the tablets based on in vivo results from dog models. Three types of controlled-release formulations were developed. The first of these was an EC-IR tablet with Eudragit L30D 55, in which the enteric coating dissolves shortly after passing through the stomach. These tablets contained the MEDI7219 peptide and permeation enhancers with other standard tablet excipients in which we optimized the ratio of Na CDC to PG permeation enhancers. The second was an EC-CR tablet with Eudragit FS30D enteric coating that dissolves at pH > 7.0; with these tablets, dissolution starts later than in the case of EC-IR tablets. In addition to MEDI7219, permeation enhancers, and other standard tablet excipients, EC-CR tablets also contained Carbopol or Eudragit RSPO to provide delayed release, so tablet contents are expected to stay longer at the site of absorption. As a third arm, we developed two OralogiK^TM^ formulations that combined enteric coating with time-delayed release to distal sites.

**Figure 4:**
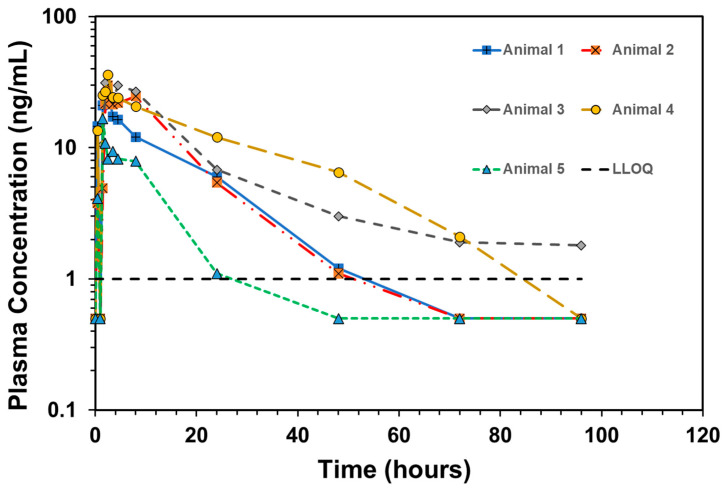
Plasma concentration–time profiles of individual animals dosed with Formulation 5. Formulation 5 = 1 ng/mL is shown as a red dashed line. All values below LLOQ were plotted as ½ LLOQ for illustration purposes.

For the EC-IR tablet formulation, we mapped 1–10 mg of MEDI7219 and 50–300 mg of permeation enhancer at ratios of 1:1, 1:2, and 2:1 Na CDC to PG (Figure 2A; Table 2, Formulations 1–9). Each tablet contained 150 mg of permeation enhancers, and a dose of two tablets was intended to achieve a 300 mg dose of permeation enhancer. Formulation 5, with 1 mg of MEDI7219 and a 300 mg total dose of permeation enhancers (100 mg of Na CDC and 200 mg of PG per dose) per animal, resulted in optimal MEDI7219 oral bioavailability, with F = 10.1%, and a minimal coefficient of variance (CV) = 26% pharmacokinetic variability at the maximum concentration (C_max_). Plasma concentration vs. time for each animal for formulation 5 is shown in Figure 4.

The next canine study was designed to further build upon the performance of Formulation 5. In this study, we evaluated formulations with 3 and 10 mg MEDI7219 peptide doses and permeation enhancers of 150, 300, and 450 mg at a 1:2 ratio of Na CDC to PG. Also evaluated in this study were groups with 300 mg permeation enhancer doses and 1:1, 1:2, and 1:3 ratios of Na CDC to PG. Tablets were enterically coated with Eudragit formula, which dissolves at pH ≥ 5.5. Increasing the dose to 3 mg while keeping the permeation enhancer dose and ratio the same as in formulation 5 produced a bioavailability of 15.5% (Formulation 15). A lower amount of permeation enhancer dose (150 mg) with a 3 mg dose (Formulation 10) also produced higher bioavailability (11.1%) than Formulation 5 (10.1%). However, both of the formulations in this study had higher pharmacokinetic variability at C_max_ (100% for Formulation 5 and 86% for Formulation 15).

In a subsequent study, the bio-performance of EC-CR (Table 3, Formulations 17–22) and OralogiK (Table 3, Formulations 23 and 24) tablet formulations of the MEDI7219 peptide were evaluated. Formulations containing Carbopol and an enteric coating of pH 5.5 demonstrated the highest bioavailability. The formulation with Carbopol 2.5% (Formulation 17) had a bioavailability of 9.9% and 80% pharmacokinetic variability at C_max_.

The mean bioavailability ranged from 1.11% to 4.78% for the two OralogiK formulations. Formulation 24 resulted in a higher bioavailability than the control immediate-release formulation, as shown by the area under the curve from time zero to infinite time (AUC_0–inf_) and F (%) (Table 4). Both Formulation 23 and 24 demonstrated an extended-release pattern compared with the IR formulation, as shown by increased time to reach maximum concentration (T_max_). T_max_ was 1.00–8.00 h for formulation 23, which is reflective of the dual-release design, 4.50–8.00 h for the delayed-release formulation 24, and 1.00–2.00 h for the immediate-release formulation. Formulation 23 had the lowest C_max_ and the lowest bioavailability of all three formulations. C_max_ was 42.3, 7.97, and 20.1 ng/mL for the immediate-release formulation, Formulation 23, and Formulation 24, respectively. AUC_0–inf_ was comparable for the immediate-release formulation and Formulation 24 but was significantly lower for Formulation 23. Both Formulation 23 and Formulation 24 had higher variability than the immediate-release formulation, but this was expected due to controlled-release behavior.

## 8. Discussion

This study reports a first-of-its-kind rational design for the optimization of peptide oral dosage forms. In an earlier study [12], we used IntelliCap capsules, a programmable drug delivery tool that can accurately target desired sites in the GI tract. Absorption of peptides was highest in the proximal colon, followed by the proximal small intestine.

We evaluated the absorption of the MEDI7219 peptide in specific locations of the GI tract, as outlined above, in dogs in the presence of permeation enhancers, and administered as immediate-release, controlled-release, and delayed-release OralogiK tablets. We hypothesized that co-release and controlled release of peptide and permeation enhancers at targeted sites in the GI tract would increase bioavailability due to increased residence time [14]. To avoid degradation in the acidic environment of the stomach, formulations were enterically coated, which avoided release in the stomach. Once the enteric coating passes through the stomach and enters the small intestine, the enteric coating begins to dissolve once the pH rises >5.5. Bile salts such as Na CDC tested in this study, act as surfactants that improve intestinal permeation by transcellular perturbation [15]. Bile salts are present in many clinically tested formulations, acting as enhancers for improved bioavailability of peptides [16]. In addition to bile salts, we also tested propyl gallate, an aromatic alcohol, as a permeation enhancer to enhance transcellular transport [17] of MEDI7219.

In this study, we focused on targeting the proximal small intestine and proximal colon. Generally, the permeability of molecules is higher in the upper portion of the small intestine than in the colonic area, primarily due to decreased surface area and tighter junctions in the colon [18,19]. This was observed in our EC-CR formulations, wherein Formulations 17 and 18 demonstrated higher bioavailability than Formulation 19 or 20. Buckley et al. also observed that efficient absorption of peptides is dependent on suitable coaction between the absorption enhancers and the co-delivered peptide [20].

OralogiK tablet formulations have the capacity to delay release, and their hydrophobic-hydrophilic compositions can be tuned to deliver the molecule at specific times and locations in the intestine. The composition and thickness of the EBL was optimized in Formulations 23 and 24 to target release in the small intestine and/or proximal colon. Formulation 24, which was designed to release in the colon, achieved significant bioavailability (approx. 5%) from the analysis of a single 8 h blood sample. Also, given the sampling schedule, we might have missed the peak plasma level.

In the current study, we kept the sampling timepoints consistent across the three formulations tested. Nevertheless, in retrospect, the blood-sampling protocol designed to characterize the pharmacokinetic profiles of formulations releasing in the upper GI tract seemed more suited to immediate and enteric-coated formulations. Adding additional sampling timepoints could have provided more information about the OralogiK formulation.

The OralogiK formulations release the peptide after a number of hours delay and at distal GI tract sites (see Table 4). For example, for the formulation targeting sustained colonic release (Formulation 24), where onset of release may only have occurred around 4-6 h, the slow-release nature of the core tablet might mean that the main corresponding phase of absorption would have only been captured by a single timepoint at 8 h, possibly underestimating C_max_, AUC, or F values due to sparse sampling, whereas the formulations releasing earlier would require more timepoints to allow adequate characterization of these key pharmacokinetic parameters. Similar considerations also exist for the delayed-release phase in formulation 23, where the dose was further split within the OralogiK tablet into two time-separated phases of release, which would be expected to lead to a comparatively lower C_max_ and perhaps two separate plasma peaks.

## 9. Conclusions

We designed MEDI7219 based on our previous site-of-absorption studies and optimized the composition of the tablets in vivo. Three site-specific, drug delivery-based tablet formulations were developed, namely, EC-IR, EC-CR, and an EBL tablet. Optimum bioavailability of 10.1% (with a 26% variability in C_max_) was achieved for EC-IR formulation, and 9.9% (80% variability in C_max_) for EC-CR formulation. The immediate-release formulation provided increased absorption of the peptide in alignment with our site-of-absorption studies showing a higher uptake in the proximal small intestine. The OralogiK formulation designed to release in the proximal colon provided a bioavailability of 4.8% (57.6% variability in C_max_) under the given blood sampling conditions. The EC-IR formulation was advanced to the clinic and was studied in the clinical trial NCT03362593.

## Figures and Tables

**Table 1:** Clinically advanced technologies for oral peptide delivery using permeation enhancers. Source: PharmaCircle. Interested readers are encouraged to refer to the National Library of Medicine’s clinical trials database (ClinicalTrials.gov) for the most recent updates. Abbreviations: FDA, U.S. Food and Drug Administration; GIPET, gastrointestinal permeation enhancement technology; GLP-1, glucagon-like peptide 1; POD, peptide oral delivery.

Company	Peptide	Technology	Permeation Enhancer Used
Emisphere Technologies	Insulin, GLP-1 analog	Eligen B12; absorption enhancers	Sodium salcaprozate (SNAC) [7]
Chiasma	Octreotide	Transient permeability enhancer technology; oil-based suspension	Sodium caprylate (C8) [8]
Enteris BioPharma	Leuprolide; difelikefalin	Peptelligence; absorption enhancers	Acylcarnitine and citric acid [9]
Merrion Pharmaceuticals	Insulin	GIPET; absorption enhancers	Sodium Caprate (C10) [10]
Oramed Pharmaceuticals	Insulin	POD; absorption enhancers and enzyme inhibitors	EDTA and/or bile salts [11]

**Table 2:** Formulations prepared for optimization of immediate-release tablet formulation. Cohorts indicate different animal studies. Cohort 1 corresponds to Figure 2A and cohort 2 corresponds to Figure 2B. CDC = chenodeoxycholate; PG = propyl gallate; PK = pharmacokinetic.

Formulation	Permeation Enhancer Content (mg)	Peptide Content (mg)	Permeation Enhancer Ratio (Na CDC:PG)	Absolute Oral Bioavailability, F, (%)	PK Variability at C_max_ (%)
Cohort 1					
1	50	10	2:1	0.3	110
2	50	10	1:2	1.8	63
3	300	10	1:2	6.3	74
4	300	10	2:1	4.8	35
5	300	1	1:2	10.1	26
6	300	1	2:1	5.0	42
7	50	1	2:1	0.1	36
8	50	1	1:2	0.0	67
9	150	5	1:1	3.2	97
10	150	3	1:2	11.1	100
Cohort 2					
11	150	10	1:2	13.5	94
12	300	3	1:3	7.5	89
13	300	10	1:2	7.5	119
14	450	3	1:2	7.5	81
15	300	3	1:2	15.5	86
16	300	3	1:1	6.6	74

**Table 3:** Formulations prepared for optimization of controlled-release tablet formulation. CDC = chenodeoxycholate; PG = propyl gallate; PK = pharmacokinetic.

Formulation	Permeation Enhancer Content (mg)	Peptide Content (mg)	Permeation Enhancer Ratio (Na CDC:PG)	Controlled-Release Polymer Type and Content (%)	Enteric Coating pH	Absolute Oral Bioavailability, F, (%)	PK Variability at C_max_ (%)
17	300	3	1:2	Carbopol 71G 2.5%	5.5	9.9	80
18				Carbopol 71G 5.0%	5.5	8.9	76
19				Carbopol 71G 2.5%	7.0	4.2	114
20				Carbopol 71G 5.0%	7.0	7.9	89
21				Eudragit RSPO 12%	5.5	2.9	98
22				Eudragit RSPO 18%	5.5	2.3	43
23		1		OralogiK formulation 1	5.5	1.1	97
24				OralogiK formulation 2	5.5	4.8	57.6

**Table 4:** PK parameters for formulations 23 and 24 in male beagle dogs. In this study, we kept the sampling timepoints consistent across the three formulations tested, which seemed more suited to IR formulations. AUC_0–inf_ = AUC from time 0 to infinite time; AUC_0–last_ = area under the curve from time 0 to last measurable concentration; CL_f_ = formation clearance rate; C_max_ = maximum concentration; F = bioavailability; NC = not calculated; T_max_ = time to maximum concentration. All pharmacokinetic parameters are shown as arithmetic mean (CV%) except T_max_, for which the median (minimum–maximum) is reported.

Formulation	t_½_ (h)	T_max_ (h)	C_max_ (ng/mL)	AUC_0–last_ (h∙ng/mL)	AUC_0–inf_ (h∙ng/mL)	AUC_0–inf_/Dose (h∙ng/mL/mg)	CL_f_ (mL/h)	F (%)
Control IR Formulation	7.16 (10.7)	1.50 (1.00–2.00)	42.3 (59.1)	321 (54.7)	439 (14.1)	439 (14.1)	2310 (13.9)	4.22 (14.1)
OralogiK Formulation 23	NC	8.00 (1.00–8.00)	7.97 (79.8)	64.5 (122.3)	116 (94.7)	116 (94.7)	14200 (63.5)	1.11 (94.7)
OralogiK Formulation 24	9.52 (20.0)	8.00 (4.50–8.00)	20.1 (114.3)	322 (106.1)	498 (57.6)	498 (57.6)	2410 (57.6)	4.78 (57.6)

## Data Availability

Data cannot be shared due to privacy restrictions.
